# Host-Mediated Antimicrobial Effects and NLRP3 Inflammasome Modulation by Caulerpin and Its Derivatives in Macrophage Models of Mycobacterial Infections

**DOI:** 10.3390/microorganisms13030561

**Published:** 2025-03-01

**Authors:** Maria Gabriella S. Sidrônio, Maria Eugênia G. Freitas, Daniel W. A. Magalhães, Deyse C. M. Carvalho, Vinícius A. B. Gonçalves, Ana Caroline M. de Queiroz Oliveira, Gisela C. Paulino, Gabriela C. Borges, Rafaelle L. Ribeiro, Natália Ferreira de Sousa, Marcus T. Scotti, Demétrius A. M. de Araújo, Francisco Jaime B. Mendonça-Junior, Kristerson R. de Luna Freire, Sandra Rodrigues-Mascarenhas, Bárbara Viviana de O. Santos, Valnês S. Rodrigues-Junior

**Affiliations:** 1 Postgraduate Program in Development and Technological Innovation in Medicines, Federal University of Paraíba (UFPB), João Pessoa 58051-900, PB, Brazil; gabriellassidronio@gmail.com (M.G.S.S.); barbara@ltf.ufpb.br (B.V.d.O.S.); 2 Laboratory of Biotechnology in Microorganisms, Biotechnology Center, Federal University of Paraíba (UFPB), João Pessoa 58051-900, PB, Brazil; m.eugeniagouveia@gmail.com (M.E.G.F.); gisela.carvalho@academico.ufpb.br (G.C.P.); gabrielacbgs@gmail.com (G.C.B.); rafalemosrib@gmail.com (R.L.R.); 3 Laboratory of Immunobiotechnology, Biotechnology Center, Federal University of Paraíba (UFPB), João Pessoa 58051-900, PB, Brazil; daniel.magalhaes.biotec@gmail.com (D.W.A.M.); deysecmc@gmail.com (D.C.M.C.); sandra@cbiotec.ufpb.br (S.R.-M.); 4 Department of Cell and Molecular Biology, Biotechnology Center, Federal University of Paraíba (UFPB), João Pessoa 58051-900, PB, Brazil; viniciusantonio548@gmail.com (V.A.B.G.); kristerson@cbiotec.ufpb.br (K.R.d.L.F.); 5 Faculty of Dentistry, Unipê, Centro Universitário—Cruzeiro do Sul Educacional, João Pessoa 58053-000, PB, Brazil; cacaguto@gmail.com; 6 Postgraduate Program in Natural and Synthetic Bioactive Products, Department of Pharmaceutical Sciences, Federal University of Paraíba (UFPB), João Pessoa 58051-900, PB, Brazil; nataliafsousa@ltf.ufpb.br (N.F.d.S.); mtscotti@gmail.com (M.T.S.); 7 Postgraduate Program in Biotechnology (Renorbio), Federal University of Paraíba (UFPB), João Pessoa 58051-900, PB, Brazil; demetrius@cbiotec.ufpb.br; 8 Laboratory of Synthesis and Drug Delivery, Department of Biological Sciences, State University of Paraíba, João Pessoa 58071-160, PB, Brazil; franciscojbmendonca@yahoo.com.br; 9 Center for Teacher Training, UACEN, University of Campina Grande, Cajazeiras 58900-000, PB, Brazil

**Keywords:** tuberculosis, drug development, caulerpin, host-directed therapy, toxicity, anti-inflammatory

## Abstract

Caulerpin, a bis-indole alkaloid isolated from *Caulerpa racemosa*, has several documented pharmacological activities, including antineoplastic and antiviral properties. This study aimed to evaluate the anti-inflammatory and anti-tubercular potentials of caulerpin and its analogues in RAW 264.7 macrophages infected with *Mycobacterium* spp. Additionally, we evaluated cytokine production and NLRP3 expression in this infection model. Toxicity tests were performed using Vero E6 and HepG2 cell lines and *Artemia salina*. Pre-incubation of RAW 264.7 cells with caulerpin and its analogues decreased internalized *M. smegmatis* and *M. tuberculosis* H37Ra. Furthermore, treatment of *M. smegmatis*-infected macrophages with caulerpin and its analogues reduced bacterial loads. Caulerpin reduced the CFU count of internalized bacilli in the *M. tuberculosis* H37Ra infection model. In addition, caulerpin and its diethyl derivative were notably found to modulate IL-1β and TNF-α production in the *M. smegmatis* infection model after quantifying pro-inflammatory cytokines and NLRP3. Caulerpin and its derivates did not affect the viability of Vero E6 and HepG2 cell lines or nauplii survival in toxicity studies. These findings demonstrate that caulerpin and its analogues exhibit anti-inflammatory activity against *Mycobacterium* spp. infection in RAW 264.7 macrophages and show promising potential for further efficacy and safety evaluation.

## 1. Introduction

Tuberculosis (TB) is a contagious infectious disease caused by the bacillus *Mycobacterium tuberculosis*, an exceptionally resilient and adaptable pathogen that primarily infects the lungs, leading to the classic syndrome of pulmonary TB. Exposure to *M. tuberculosis* bacilli leads to elimination or persistence of the pathogen based on the host’s immune response. From the clinical standpoint, the disease spectrum ranges from an asymptomatic and non-transmissible state, known as latent TB infection, to an active and highly transmissible TB disease [1,2].

Epidemiologically, TB remains a major global public health problem. TB returned to being the leading global cause of death from a single infectious agent in 2023, three years after it was replaced by the coronavirus disease (COVID-19), resulting in almost twice as many deaths compared to HIV/AIDS. An estimated 10.8 million people fell ill with TB globally in 2023, a further increase from 10.7 million in 2022. This continued rise reflects the ongoing after-effects of disruptions to TB services during the worst years of the COVID-19 pandemic (2020 and 2021) [3].

The emergence of drug-resistant variants of *M. tuberculosis* has made treatment and eradication of TB significantly more difficult. Drug-resistant *M. tuberculosis* strains which occur in both hospital and community settings exhibit varying degrees of drug resistance, such as resistance to rifampicin (RR), multi-drug resistance (MDR), and extensively drug-resistant (XDR) forms [4,5]. The estimated global annual number of people who developed RR-TB or MDR-TB remained relatively flat between 2020 and 2023, after a slow downward trend between 2015 and 2020 [3].

Antibiotic resistance is indeed a critical issue that threatens global public health, and the lack of new antibiotics with novel mechanisms of action only exacerbates the problem. While the discovery and development of new antibiotics is essential, focusing on multifaceted therapeutic approaches is crucial to addressing this challenge effectively [6].

Host-directed therapy (HDT) for TB involves various biological, pharmacological, and immunological interventions. The aim is to alter the host’s response to *M. tuberculosis* infection with the goal of boosting the protective immune responses, suppressing the overwhelming inflammatory responses, enhancing the ability of the host to maintain a state of homeostasis, and improving the efficacy of chemotherapy [7,8]. Despite advances in HDT, many complications remain regarding the synthesis of new compounds to integrate into this therapeutic methodology. Some of the main challenges are the side effects of these new compounds, in addition to the high costs and challenges in replicating results. Natural products represent a promising option in the search for new compounds that can be incorporated into this therapeutic approach, as they offer mild modulatory effects on the host’s immune responses, with less immunopathological damage and lower costs [9].

Natural products are often complex mixtures of bioactive compounds which can act on multiple biological targets, providing a broad-spectrum effect. This polypharmacological action is particularly useful in combating complex diseases like TB. The use of natural products in combination therapies is also a promising strategy, as the combination of different bioactive compounds may reduce the likelihood of resistance development and enhance therapeutic efficacy. Marine microorganisms offer great potential for developing new antibiotics, possibly because these microorganisms have evolved unique metabolic and physiological capabilities to ensure survival in extreme ocean habitats [10,11].

**Figure 1:**
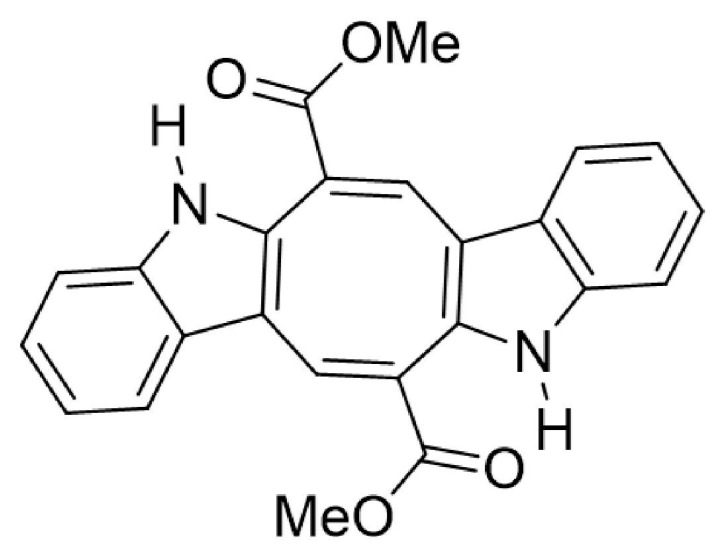
Chemical structure of CP.

Macroalgae have gained attention as valuable bioactive compound sources with various therapeutic properties, including anti-inflammatory effects [12]. Caulerpin (CP) (Figure 1), a bis-indole alkaloid isolated from *Caulerpa racemosa* and *Caulerpa serrulata* (green macroalgae native to southwestern Australia and the Mediterranean Sea), has several pharmacological activities already described, such as neoplastic and antiviral activities [13,14,15].

CP showed anti-inflammatory effects in a murine model of peritonitis and ulcerative colitis, providing an improvement in the disease activity index, in addition to attenuating colon shortening and damage [16]. Furthermore, anti-inflammatory effects of CP were observed against chronic infection and disorders related to the pathogenesis of *Helicobacter pylori* [17]. There are currently no studies reporting anti-inflammatory and host-mediated effects of CP against *M. tuberculosis*. In this work, we assessed the anti-inflammatory activities of CP and derivative molecules against mycobacteria using infection models with murine cells. Furthermore, we focused on understanding the mechanisms by which these drugs elicited their effects.

## 2. Materials and Methods

### 2.1. CP Extraction and Isolation

CP was extracted using a previously described methodology [15]. *C. racemosa* was collected in the city of Pitimbu, Paraíba State, in the northeastern region of Brazil. The dried material was submitted to exhaustive extraction with EtOH, followed by its concentration in a rotary evaporator, obtaining the respective crude extract. A portion (30 g) of the crude extract of *C. racemosa* was submitted to open-column adsorption chromatography (CC) in silica gel (Merck, Darmstadt, Germany; Ø µm 63–200) and hexane and CH_2_Cl_2_ solvents, with elution orders of hexane, hexane: CH_2_Cl_2_ (1:1), hexane: CH_2_Cl_2_ (8:2), and CH_2_Cl_2_. The fractions were concentrated on a rotary evaporator and pooled according to TLC analysis. This procedure provided 2.1 g (7%) of pure CP.

### 2.2. Preparation of Derivatives

**Figure 2:**
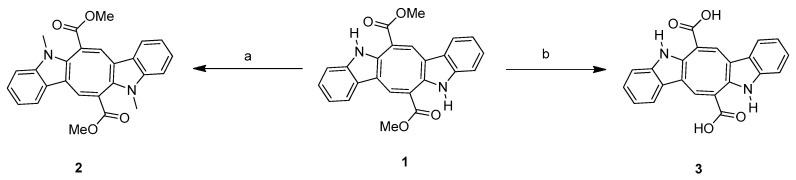
Both 2 and 3 are derivatives of CP (**1**). Reagents and conditions: (a) **2**: KOH, Me_2_SO_4_; MeOH, acetone/room temperature, magnetic stirring. (b) **3**: KOH, acetonitrile: water, 60 °C, magnetic stirring [15].

**Figure 3:**
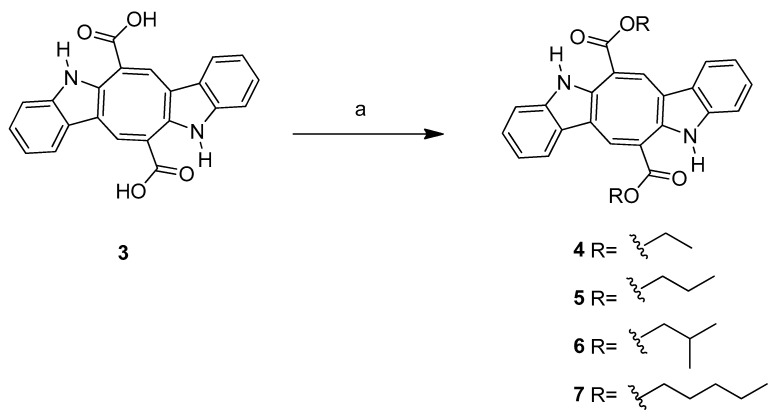
Objects 4–7 are derivatives of CP (**1**). Reagents and conditions: (a) SOCl_2_, ethyl alcohol (**4**), propyl alcohol (**5**), isobutyl alcohol (**6**), amyl alcohol (**7**), 60 °C, magnetic stirring.

The natural CP (**1**) product belongs to a family of bis-indolic alkaloids characterized by an additional eight-membered ring connecting two indolic rings directly incorporated with a carbonyl group. Several analogs were proposed for further investigation due to the promising biological activities of compound (**1**). The *N*-methyl derivative (**2**) and caulerpinic acid (CA) (**3**) were synthesized from CP (**1**) following the methodology described in [15]. *N*-Methylation was performed using methyl sulfate in a basic medium, and CA (**3**) was obtained via basic hydrolysis of compound **1** (Figure 2). Using CA (**3**) as the starting material, diethyl (DE) (**4**), dipropyl (DP) (**5**), diisobutyl (**6**), and diamyl (**7**) esters were synthesized through esterification in the presence of thionyl chloride (Figure 3) [18].

### 2.3. General Method for Obtaining 4–7 Analogs

First, 0.013 mL of thionyl chloride (SOCl_2_) (0.1839 mmol) was added dropwise at room temperature to a solution of CA (**2**) (0.0814 mmol, 30 mg) in *n*-propyl alcohol (5 mL). The temperature was then raised to 60 °C, and the reaction mixture was stirred magnetically and monitored by TLC until complete consumption of the starting material (6 h). The mixture was evaporated under reduced pressure, suspended in distilled water, and extracted with ethyl acetate (3 × 20 mL). The organic phase was combined, treated with anhydrous sodium sulfate (Na_2_SO_4_), filtered, and concentrated under reduced pressure. The resulting material was subjected to preparative thin-layer chromatography (PTLC) using hexane and ethyl acetate as eluents (*n*-Hexane:AcOEt—80:20), yielding the corresponding purified derivatives [18].

*(6E,13E)-diethyl 5,12-dihydrocycloocta[1,2-b:5,6-b′]diindole-6,13-dicarboxylate* (**4**). Yield: (23.4 mg) 78%; aspect: red solid; solubility: CH_2_Cl_2_; molecular formula: C_26_H_22_N_2_O_4_; molar mass: 426.46 g/mol. IR (KBr) ν_max_/cm^−1^: 3382, 3055, 2997, 2952, 2925, 2852, 1687, 1631, 782. ^1^H NMR (200 MHz, CDCl_3_, ppm) δ: 9.29 (s. 1H); 8.03 (s. 1H); 7.42 (d. 1H); 7.35 (m. 4H); 7.17 (m, 4H); 7.08 (m, 4H); 4.37 (q, 2H); 1.44 (m, 3H); 1.41 (t, 2H). ^13^C NMR (50 MHz, CDCl_3_, ppm) δ: 166.4; 142.5; 137.8; 133.1; 128.3; 125.8; 123.4; 120.7; 118.6; 118.0; 112.4; 111.6; 61.7; 14.4.

*(6E,13E)-dipropyl 5,12-dihydrocycloocta[1,2-b:5,6-b′]diindole-6,13-dicarboxylate* (**5**). Yield: (22.5 mg) 75%; aspect: orange solid; solubility: CH_2_Cl_2_; molecular formula: C_28_H_26_N_2_O_4_; molar mass: 454.52 g/mol. IR (KBr) ν_max_/cm^−1^: 3998, 3056, 3032, 2964, 2926, 2898, 2876, 2852, 1679, 1611, 1460, 1415, 1321, 1260, 1180, 1065, 949, 769, 731, 544. ^1^H NMR (200 MHz, CDCl_3_, ppm) δ: 9.31 (s, 1H); 8.04 (s, 1H); 7.41 (d, 1H); 7.39 (d, 1H); 7.28 (d, 2H); 7.17 (m, 2H); 7.08 (m, 2H); 4.26 (t, 2H); 1.75 (m, 2H); 1.08 (t, 3H); 1.05 (t, 3H). ^13^C NMR (50 MHz, CDCl_3_, ppm) δ: 166.4; 142.5; 137.5; 133.2; 128.3; 125.8; 123.4; 120.8; 118.0; 112.4; 111.6; 67.2; 22.1; 10.7.

*(6E,13E)-diisobutyl 5,12-dihydrocycloocta[1,2-b:5,6-b′]diindole-6,13-dicarboxylate* (**6**). Yield: (21 mg) 70%; aspect: brown solid; solubility: CH_2_Cl_2_; molecular formula: C_30_H_30_N_2_O_4_; molar mass: 482.22 g/mol. IR (KBr) ν_max_/cm^−1^: 3979, 3056, 2961, 2891, 2871, 16,798, 1626, 1481, 1456, 1415, 1250, 1215, 1176,1155, 1031, 769, 741, 668, 609, 482. ^1^H NMR (200 MHz, CDCl_3_, ppm) δ: 9.35 (s, 1H); 8.06 (s, 1H); 7.40 (d, 1H); 7.27 (d, 1H); 7.18 (m, 3H); 7.07 (m, 3H); 4.09 (d, 2H); 2.05 (m, 1H); 1.03 (d, 6H). ^13^C NMR (50 MHz, CDCl_3_, ppm) δ: 166.4; 142.5; 137.8; 133.2; 128.3; 127.4; 123.4; 120.7; 118.0; 112.4; 111.6; 71.7; 27.9; 19.3.

*(6E,13E)-dipentyl 5,12-dihydrocycloocta[1,2-b:5,6-b′]diindole-6,13-dicarboxylate* (**7**). Yield: (20.1 mg) 67%; aspect: orange solid; solubility: CH_2_Cl_2_; molecular formula: C_32_H_34_N_2_O_4_; molar mass: 510.62 g/mol. IR (KBr) ν_max_/cm^−1^: 3401, 3372, 3058, 2952, 2932, 2855, 1680, 1628, 1459, 1415, 1321, 1259, 1180, 1158, 1056, 732. ^1^H NMR (200 MHz, CDCl_3_, ppm) δ: 9.32 (s, 1H); 8.03 (s, 1H); 7.40 (d, 4H); 7.28 (m, 4H); 7.18 (d, 4H); 7.08 (m, 4H); 4.32 (t, 2H); 4.29 (t, 2H); 4.25 (t, 2H); 1.81 (m, 2H); 1.78 (m, 2H); 1.41 (m, 4H); 0.94 (t, 3H). ^13^C NMR (50 MHz, CDCl_3_, ppm) δ: 166.4; 142.5; 137.8; 133.2; 128.3; 125.8; 123.4; 120.7; 118.0; 112.4; 111.6; 67.8; 28.4; 28.3; 22.5; 14.1.

### 2.4. Bacteria and Cell Lines

*Mycobacterium smegmatis* mc^2^155 and *M. tuberculosis* H37Ra laboratory strains were maintained in 7H9 media supplemented with Tween-80 (0.05%) and 10% oleic acid-albumin-dextrose-catalase (OADC). *M. smegmatis* and *M. tuberculosis* inocula were prepared as previously described [19]. The murine macrophage cell line RAW 264.7, renal epithelial cells Vero E6, and human liver cells HepG2, obtained from the Cell Bank of Rio de Janeiro, were cultured in DMEM media (Dulbecco’s Modified Eagle Medium, Gibco, Grand Island, NY, USA) supplemented with 10% inactivated fetal bovine serum (FBS) and 1% antibiotics (penicillin–streptomycin). The cells were maintained in culture flasks at 37 °C in a humidified atmosphere with 5% CO_2_.

### 2.5. Evaluation of the Direct Effects of CP and Analogues on RAW 264.7 Cell Viability

The direct effects of CP and its analogues on RAW 264.7 viability were evaluated using the MTT method [19]. RAW 264.7 cells were seeded at 1 × 10^4^ cells/well (for 24 h incubation) and 5 × 10^3^ cells/well (for 48 h incubation) and incubated overnight to allow adhesion. The culture medium was carefully aspirated and replaced with 100 μL DMEM plus 100 μL of test compound solutions, yielding final concentrations ranging from 3.25 to 30 µM (DMSO 1%, *v*/*v*). After 24 or 48 h of exposure to test compounds, cells were incubated with MTT (0.5 mg/mL) for 3 h. The resulting formazan crystals were dried, dissolved in DMSO, and absorbance was measured at 570 nm using a microplate reader (EL800, BioTek, Winooski, VT, USA). The precipitated purple formazan crystals were directly proportional to the number of metabolically active viable cells. The cell viability was then calculated as: Cell viability (%) = (Sample absorbance/Control absorbance) × 100. Data were expressed as mean of cell viability ± standard error of the mean from three to four independent experiments performed in triplicate.

### 2.6. M. smegmatis Susceptibility Investigation

CP concentrations and its analogues which did not affect RAW 264.7 cell viability were used in this experiment. The evaluation of *M. smegmatis* mc^2^155 susceptibility to the test compounds was performed by enumeration of colony forming units (CFU) [19,20]. CP, CA, DE, and DP were diluted in Middlebrook 7H9 medium containing 10% OADC at concentrations ranging from 1.85 to 15 µM. Rifampicin (RIF) at 31 µM was used as a positive control. The final DMSO concentration in all wells was 2.5%. *M. smegmatis* suspension was diluted in Middlebrook 7H9 with 10% OADC to achieve an optical density of 0.001 at 595 nm, and 100 μL was then added to each well. The plates were covered, sealed with parafilm, and incubated at 37 °C. The aliquots were removed from each well after 24 h of incubation, serially diluted, and plated on Luria Bertani (LB) agar. The plates were incubated at 37 °C for 3 days before CFU counting. CFU numbers were expressed as log_10_ (CFU/mL).

### 2.7. Evaluation of the Therapeutic Potential of CP and Analogues in M. smegmatis-Infected RAW 264.7 Cells

For the first in vitro infection test with *M. smegmatis*, RAW 264.7 were seeded in 96-well culture plates at a density of 1 × 10^4^ cells/well in DMEM medium (with 10% FBS, without antibiotics) and incubated overnight at 37 °C with 5% CO_2_ [20]. Infection of RAW 264.7 cells with *M. smegmatis* mc^2^155 was performed at a multiplicity of infection of 1:1 for 2 h at 37 °C with 5% CO_2_. The medium with non-internalized bacteria was removed, and infected cells were then treated with the tested substances in DMEM: CP (15 µM, 7.5 µM and 3.25 µM), CA (30 µM, 15 µM, 7.5 µM and 3.25 µM), DE (15 µM, 7.5 µM and 3.25 µM), and DP (15 µM, 7.5 µM and 3.25 µM). Macrophages were lysed after 24 h with 0.1% Triton–X100, and these suspensions were diluted and plated on LB. CFU was determined after incubation of plates for 3 days at 37 °C. CFU numbers were expressed as log (CFU/well).

### 2.8. Evaluation of the Preventive Effects of CP and Analogues in M. smegmatis-Infected RAW 264.7 Cellss

For the second in vitro cellular infection test with *M. smegmatis*, RAW 264.7 were seeded in 96-well culture plates at a density of 1 × 10^4^ cells/well in DMEM medium (with 10% FBS, without antibiotics) and incubated overnight at 37 °C with 5% CO_2_. Cells were pre-incubated with CP (15 μM), CA (30 μM), DE (15 μM), and DP (15 μM) for 8 h in DMEM medium. Infection of RAW 264.7 cells with *M. smegmatis* was performed at a multiplicity of infection of 1:1 for 2 h at 37 °C with 5% CO_2_ [20]. Infected RAW 264.7 were then lysed with 0.1% Triton–X100, and these suspensions were diluted and plated on LB agar. Remaining wells were then re-treated with the tested substances in DMEM medium. Macrophages were lysed after 12 h, and these suspensions were diluted and plated on LB prior to CFU counting. CFU/well was determined as previously described in the infection experiments using macrophages and *M. smegmatis*.

### 2.9. Quantification of Cytokine Production

IL-1β, IL-10, and TNF-α levels in the culture supernatant were determined by sandwich enzyme-linked immunosorbent assay as per the manufacturer’s instructions. Optical density was read at 450 nm using a microplate spectrophotometer (microplate reader EL800, BioTek, Winooski, VT, USA).

### 2.10. NLRP3 Evaluation by Flow Cytometry

Cells were placed in a 96-well plate (U bottom) 24 h after treatment in culture. Then, cells were fixed by Cytofix (BD Cytofix™, Franklin Lakes, NJ, USA) and permeabilized by Perm Buffer (BD Phosflow™, San Diego, CA, USA) for 30 min each. Cells were subsequently labeled with an unconjugated anti-NLRP3 antibody, according to the manufacturer’s instructions (BD Biosciences, San Diego, CA, USA). Finally, anti-rat IgG2a (PE) was added for NLRP3 analysis and cells were suspended in PBS and evaluated in a BD Accuri C6 Plus flow cytometer.

### 2.11. M. tuberculosis Susceptibility Investigation

Next, CP, CA, and DE were diluted in Middlebrook 7H9 plus 10% OADC for the cellular susceptibility assay with *M. tuberculosis* H37Ra to yield concentrations up to 15 µM, 30 µM, and 15 µM, respectively. RIF at 0.03 µM was used as a positive control. The final DMSO concentration in all wells was 2.5%. The *M. tuberculosis* suspension was diluted in 7H9 plus 10% OADC to achieve an optical density of 0.006 at 595 nm, and 100 μL was then added to each well. Plates were covered, sealed with parafilm, and incubated at 37 °C for 48 h. After incubation, aliquots were removed, diluted, and plated in Middlebrook 7H10 agar plus 10% OADC. Plates were incubated at 37 °C for 4 weeks, and CFU were counted. CFU numbers were expressed as log_10_(CFU/mL).

### 2.12. Evaluation of the Therapeutic Potential of CP and Analogues in M. tuberculosis-Infected RAW 264.7 Cells

For the infection test with *M. tuberculosis* H37Ra, RAW 264.7 were seeded in 96-well culture plates at a density of 4 × 10^3^ cells/well in DMEM medium (with 10% FBS, without antibiotics) and incubated overnight at 37 °C with 5% CO_2_ [20]. Infection of RAW 264.7 cells with *M. tuberculosis* was performed at a multiplicity of infection of 1:1 for 3 h at 37 °C with 5% CO_2_. The medium with non-internalized bacteria was removed and infected cells were then treated with the tested substances in DMEM:CP (15 µM, 7.5 µM, and 3.25 µM), CA (30 µM), and DE (15 µM) for 24 h and CA (30 µM and 15 µM) and DE (15 µM) for 48 h. Macrophages were lysed after incubations with 0.1% Triton–X100, and these suspensions were diluted and plated on Middlebrook 7H10 plus 10% OADC. Plates were incubated for 4 weeks at 37 °C and data were expressed as log_10_(CFU/well).

### 2.13. Evaluation of the Preventive Effects of CP and Analogues in M. tuberculosis-Infected RAW 264.7 Cells

For the second cellular infection test with *M. tuberculosis* H37Ra, RAW 264.7 cells were seeded in 96-well culture plates at a density of 4 × 10 ^3^ cells/well in DMEM medium (with 10% FBS, without antibiotics) and incubated overnight at 37 °C with 5% CO_2_. Cells were pre-incubated with the CP (15 µM), DE (15 µM), and CA (30 µM) compounds. Infection of RAW 264.7 cells with *M. tuberculosis* was performed at a multiplicity of infection of 1:1 for 3 h at 37 °C with 5% CO_2_ [20]. Infected RAW 264.7 were then lysed with 0.1% Triton-X100, and suspensions were diluted and plated on Middlebrook 7H10 agar plus OADC. Remaining wells were then re-treated with the test substances in DMEM medium. Macrophages were lysed after 12 h and plated on Middlebrook 7H10 containing OADC prior to CFU counting. CFU/well was determined as previously described in the infection experiments using macrophages and *M. smegmatis*.

### 2.14. Molecular Docking Simulations

Molecular docking simulations belonging to the Structure-Based Drug Design (SBDD) approach were performed with the aim of contributing to elucidating the anti-inflammatory action mechanism of the CP and DE compounds through their binding affinity to the targets involved in this effect, which correspond to the Cyclooxygenase 1 (COX-1) enzymes, referring to a model obtained from the Alpha Fold Protein Structure Data Base Platform (https://alphafold.com/, accessed on 17 December 2024) under the code: AF-Q3UZ23-F1-v4, obtained from the primary sequence from the UniProt database (https://www.uniprot.org/uniprotkb/Q9Y2T6/entry, accessed on 17 December 2024) and Cyclooxygenase 2 (COX-2) in complex with selective inhibitor SC-558 (PDB: 6COX) with a resolution of 2.80 Å [21]. For this, molecular docking simulations were performed with the 3D crystallographic structures mentioned, which were obtained from the Protein Data Bank (PDB) Library (https://www.rcsb.org/pdb/home/home.do, accessed on 22 November 2024) [22].

The test compounds under study were drawn in the Marvin Sketch v. 21.18 program (https://chemaxon.com/marvin, accessed on 22 November 2024) and saved as an .sdf file. Then, the energy minimization of the compounds was performed in Spartan v.1.14 (https://www.wavefun.com/spartan-version-history, accessed on 22 November 2024) software in order to obtain the most stable conformation, which was performed using geometry optimization through the Molecular Mechanics (MM) method with the Merck Molecular Force Field (MMFF) methodology at the ground state, followed by energy minimization performed using the Austin Model 1 (AM1) method. Prior to the geometry optimization steps and energy minimization, a conformational search was performed to obtain the most stable conformation. In the Spartan 14 program, after inserting the compound, it was automatically converted into a 3D conformation.

After obtaining the three-dimensional (3D) structure and consequently obtaining the most stable conformation, a molecular docking simulation was performed with all water molecules being removed from the crystal structure. A template was then created between the enzyme and the co-crystallized ligand to demarcate the active site of the macromolecule. The procedure was continued by inserting the test molecule, and finally the molecular docking simulation was performed. Redocking was performed prior to the docking simulation, which corresponds to the root mean square deviation (RMSD) calculated from the poses, indicating the degree of reliability of the fit. The RMSD provides the connection mode close to the experimental structure and is considered successful if the value is less than 2.0 Å [23].

The active site for the COX-1 (AlphaFold Model) enzyme (which does not present co-crystallized ligand) was identified by obtaining cavities using the search algorithm of the Molegro Virtual Docker 6.0 (MVD) software, targeting the Arginine 376 residue, which was visualized in works developed by El-Din and Barseen (2016) [24]. In addition, residues in positions 207 to 378 are related to stabilization of the radical intermediate during catalysis, and the serine 530 residue is an irreversible inhibition site of the enzyme [25], being in a displaced position with the number of serine 532 for being an AlphaFold model [26], as the preparation of these 3D structures was also previously performed by visualizing and checking the 3D structure in PyMol 2.0 Schrödinger LLC (2016) software in order to verify the structure integrity. Missing hydrogens were added with the PyRX tool (https://pyrx.sourceforge.io/, accessed on 22 November 2024) [27]. The protein was subsequently subjected to energy minimization using UCSF Chimera 1.18 software (2024) [28] with the Add Charges function to correct charges and ionization states, contribute to relaxing possible structural tensions, and remove loop regions, and the structure was modeled in order to add missing residues.

### 2.15. Molecular Docking Simulation and Visualization of Interactions

Molegro Virtual Docker v.6.0.1 (MVD) software was used with its predefined parameters to perform the molecular docking simulations [29]. A complex ligand was used to define the active site. Then, the compounds were imported to analyze the system stability through the interactions identified with the enzyme active site, taking the energy value of the MolDock Score and PLANTS Score algorithms as reference [29]. The MolDock SE (Simplex Evolution) algorithm was used with the following parameters: A total of 20 runs with a maximum of 2000 interactions using a population of 50 individuals, 2000 minimization steps for each flexible residue, and 2000 global minimization steps per run. The MolDock Score (GRID) scoring function was used to calculate the docking energy values. A GRID was set at 0.3 A, and the search sphere was set at 15 A in radius. Then, internal electrostatic interactions, internal hydrogen bonds, and *“sp2-sp2*” torsions were evaluated for the ligand energy analysis [29]. The Discovery Studio Visualizer v20.1.0.19295—BIOVIA (2020) software (https://discover.3ds.com/discovery-studio-visualizer-download, accessed on 22 November 2024) was used to visualize the interactions and obtain the molecular docking figures.

### 2.16. Cytotoxicity Evaluation of CP and Its Analogues on Vero E6 and HepG2 Cell Lines

Cytotoxicity was evaluated using the MTT method [30]. For this purpose, two cell lines were tested: Vero E6 and HepG2. Both cell lines were seeded at 1 × 10^4^ cells/well in 96-well microtiter plates and incubated overnight to adhere. The culture medium was carefully aspirated and replaced with 100 µL DMEM plus 100 µL of test compound solutions (CP, CA, and DE), yielding final concentrations ranging from 30 to 120 µM (1% DMSO, *v*/*v*). Cells were incubated with test compounds for 24 h at 37 °C in a 5% CO_2_ atmosphere. MTT solution (0.5 mg/mL) was subsequently added, and cells were incubated for an additional 3 h. The resulting formazan crystals were dried at room temperature for 24 h and dissolved in DMSO. Absorbance was measured at 570 nm (microplate reader EL800, BioTek, Winooski, VT, USA). Cell viability was calculated as previously described in the RAW 264.7 cell viability assay section.

### 2.17. Artemia Salina Toxicity Test

*Artemia salina* (brine shrimp) survival was determined after incubation with CP and its analogues following the method described by Magalhães et al. [31]. *A. salina* cysts were purchased from a local aquarium store and hatched (0.5 g cysts/L) in modified artificial seawater (35 g/L sea salt) supplemented with dried yeast (6 mg/L). Aeration was provided using an aquarium air pump connected to a line extending to the bottom of the hatching vessel. After 2 days incubation (in a 23–25 °C room), nauplii were collected using a Pasteur pipette after attracting the organisms to one side of the vessel with a light source. Test compounds were serially diluted 2-fold (ranging from 30 to 120 µM) in 96-well microplates containing 100 µL of 0.9% NaCl solution with dried yeast (6 mg/L). All experiments were performed in quadruplicate with a final DMSO concentration of 2.5%. Vehicle control wells (with 2.5% DMSO) were included in the experiment. A suspension containing 8–12 nauplii in 100 µL was added to each well, and the plates were incubated at 23–25 °C for 24 h. The number of live and dead (non-motile) nauplii in each well was then counted under a microscope. Nauplii were considered dead if they remained immobile for 10 s under light exposure. The survival percentage for each test group was calculated relative to the control group (2.5% DMSO), which was set as 100%.

### 2.18. Statistical Analysis

Statistical analysis was performed using GraphPad Prism 5.0 (GraphPad Software, San Diego, CA, USA). Data from bacterial susceptibility assays, infection models, *A. salina* survival, and cytotoxicity assays were evaluated by one-way analysis of variance (ANOVA), followed by Dunnett’s post hoc test. Cytokine data were analyzed by one-way analysis of variance (ANOVA), followed by Tukey’s test. Differences were considered significant at the 95% level of confidence.

### 2.19. Flow Cytometry Data Analysis

First, macrophages were visualized using size (FSC) and granularity (SSC) parameters, and a gate was performed in the cell population. The macrophages were then analyzed by measuring the median fluorescence intensity (MFI) corresponding to antibody labeling. MFI data were normalized to the control group, which was set to 100%. These data were evaluated using FlowJo software version 10.

## 3. Results

### 3.1. Evaluation of the Direct Effects of CP and Its Analogues on RAW 264.7 Cell Viability

**Figure 4:**
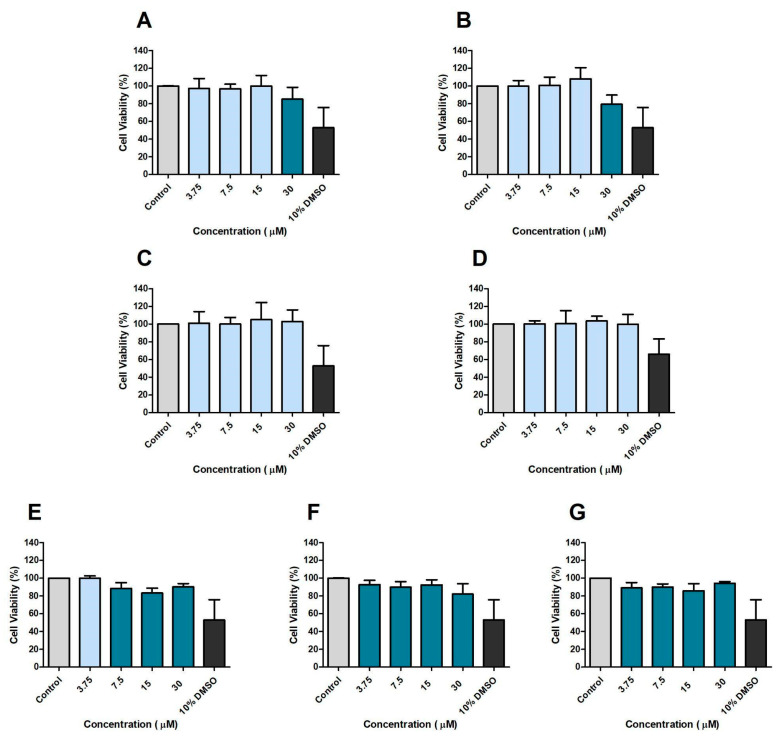
Effects of CP and analogues on RAW 264.7 cells’ viability after 24 h incubation. CP (**A**), DE (**B**), CA (**C**), DP (**D**), Diisobutyl (**E**), *N*-methyl (**F**), and Diamyl (**G**). Control: 0.5% DMSO-treated wells, considered as 100% of cell viability.

Prior to infection experiments using macrophages (RAW 264.7 cells), the direct effect of CP and its analogues on RAW 264.7 viability was assessed after 24 h incubation (Figure 4). CP and DE affected cell viability at the concentration of 30 μM (Figure 4A,B). CA and DP did not affect viability at any of the concentrations tested (Figure 4C,D). Diisobutyl affected cell viability at the three highest concentrations assessed (Figure 4E). *N*-methyl and diamyl affected cell viability at all concentrations assessed (Figure 4F,G).

**Figure 5:**
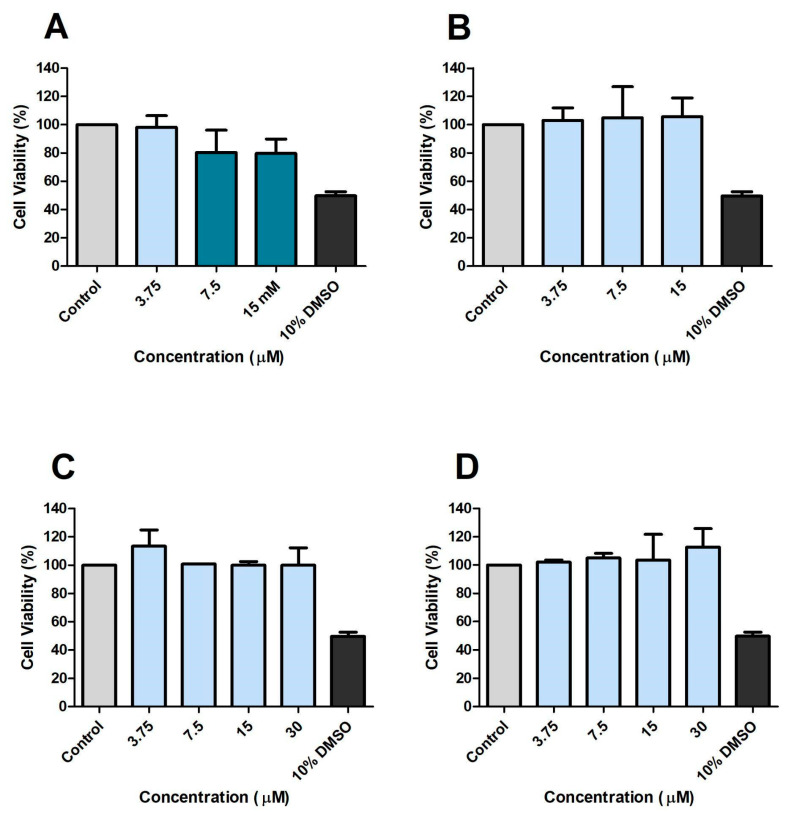
Effects of CP and analogues on RAW 264.7 cells’ viability after 48 h incubation. CP (**A**), DE (**B**), CA (**C**), and DP (**D**). Control: 0.5% DMSO-treated wells, considered as 100% of cell viability.

CP concentrations and each analogue that did not reduce macrophage viability by more than five percent compared to untreated controls after 24 h of treatment were further assessed in 48 h incubation experiments (Figure 5). CP affected cell viability at the 2 highest concentrations tested, 15 and 30 μM (Figure 5A). DE, CA, and DP did not affect cell viability at any concentration evaluated (Figure 5B–D). These concentrations were then selected for macrophage infection experiments to avoid false-positive results. RAW 264.7 cells treated with 10% DMSO served as a positive control for cell death.

### 3.2. M. smegmatis Susceptibility Testing

**Figure 6:**
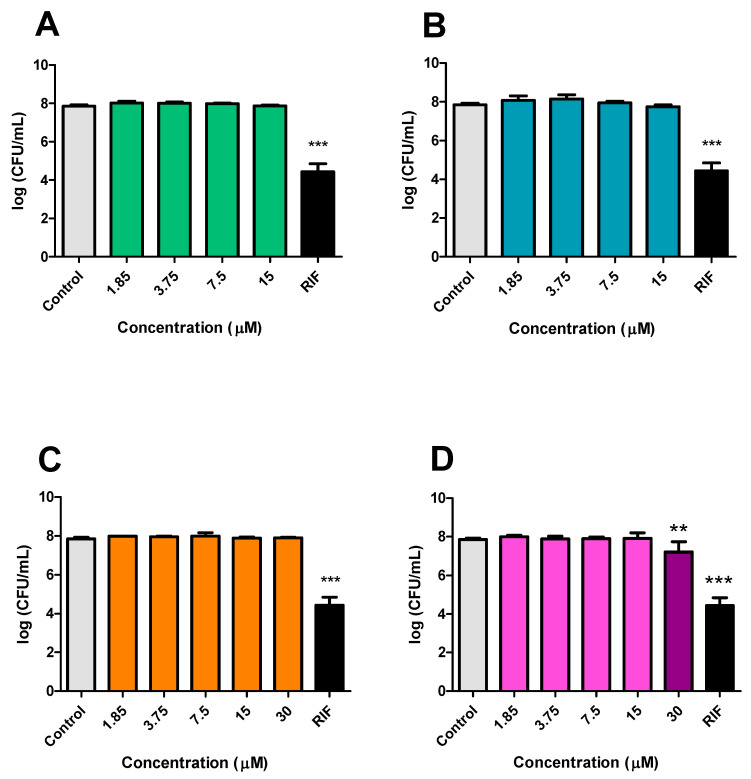
Evaluation of the effects of CP and its analogues on the viability of *M. smegmatis* bacillus after 24 h incubation. CP (**A**), DE (**B**), CA (**C**), and DP (**D**). Control: 2.5% DMSO-treated group. *** *p* < 0.001 and ** *p* < 0.01 compared to the control. Data were evaluated by ANOVA, followed by Dunnett’s post hoc test, using GraphPad Prism 5.0.

*M. smegmatis* was not susceptible to CP, DE, and CA at the tested concentrations, indicating that the inhibitory concentrations are higher than 15 μM for CP (Figure 6A) and DC (Figure 6B) and higher than 30 μM for CA (Figure 6C). In contrast, DP at 30 μM reduced *M. smegmatis* viability by 0.639 log_10_ CFU (Figure 6D). The antituberculosis drug RIF at 31 μM was also evaluated, and it reduced the CFU count by 3.41 log_10_ after 24 h of incubation.

### 3.3. Infection Experiments Using Macrophages and M. smegmatis

**Figure 7:**
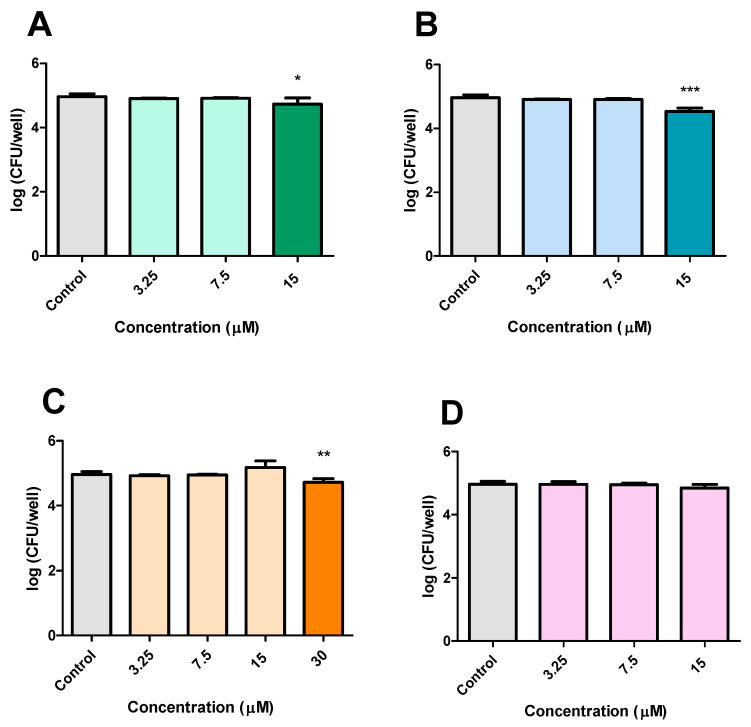
Effects of CP and its analogues in macrophages infected with *M. smegmatis*. CP (**A**), DE (**B**), CA (**C**), and DP (**D**). Control: 0.5% DMSO-treated group. *** *p* < 0.001 ** *p* < 0.01 and * *p* < 0.05 compared to the control group. Data were evaluated by ANOVA, followed by Dunnett’s post hoc test, using GraphPad Prism 5.0.

The first infection experiment investigated the antimycobacterial activity of CP, DE, CA, and PC against *M. smegmatis* within RAW 264.7 macrophages. CP (15 μM) and DE (15 μM) reduced the bacterial burden by 0.230 log_10_ and 0.430 log_10_ CFU after 24 h of treatment, respectively, compared to untreated controls (Figure 7A,B). CA (30 μM) also demonstrated antibacterial activity, reducing bacterial counts by 0.243 log_10_ (Figure 7C). In contrast, DP showed no inhibitory effect on intracellular *M. smegmatis* growth in RAW 264.7 macrophages (Figure 7D).

**Figure 8:**
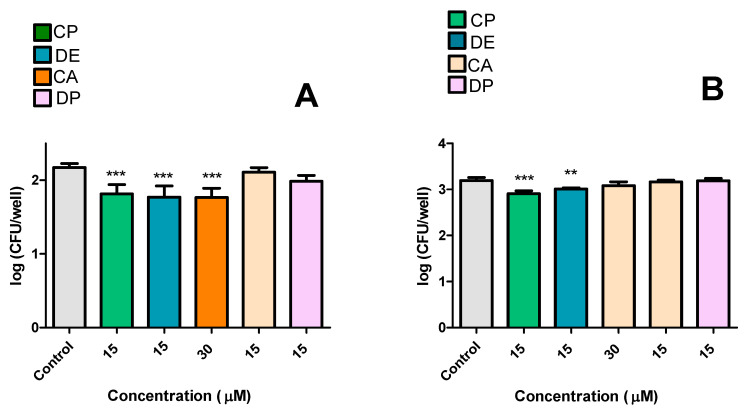
Effects of pre-incubation of CP and its analogues with RAW 264.7 infected with *M. smegmatis.* (**A**): immediately after 2 h of infection, (**B**): 12 h after infection. Control: 0.5% DMSO-treated group. *** *p* < 0.001 and ** *p* < 0.01 compared to the control group. Data were evaluated by ANOVA, followed by Dunnett’s post hoc test, using GraphPad Prism 5.0.

The second experimental design aimed to evaluate the effects of pre-incubating RAW 264.7 cells with CP, DE, CA, and DP. The results demonstrated that CP and DE at 15 μM significantly reduced bacterial loads by 0.360 log_10_ and 0.402 log_10,_ respectively. CA at 30 μM also showed a reducing effect when incubated before infection, decreasing CFU counts by 0.407 log_10_ (Figure 8A). At a later time point (12 h after infection), re-treatment with CP and DE at 15 μM further reduced CFU counts by 0.287 log_10_ and 0.182 log_10_, respectively (Figure 8B).

### 3.4. Immunomodulatory Activity of CP and DE During M. smegmatis Infection of RAW 265.7 Cells

**Figure 9:**
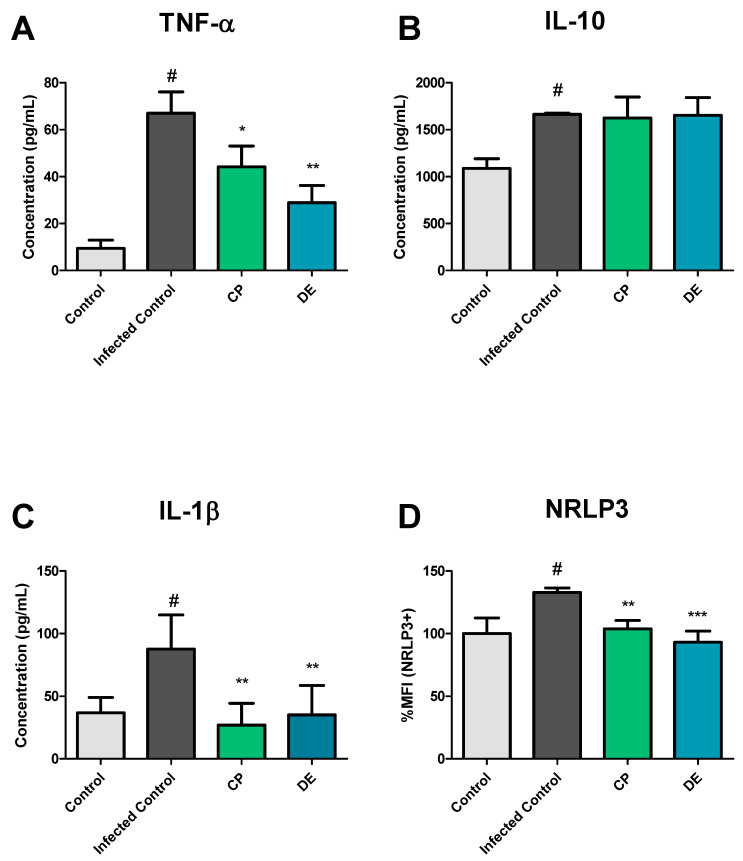
Effects of CP and DE on cytokine levels and inflammasome (NLRP3) expression during infection of RAW 264.7 cells with *M. smegmatis* after 24 h incubation. Control: RAW 264.7 cells only; Infected Control: RAW 264.7 cells infected with *M. smegmatis* (MOI 1:1). CP: infected cells treated with CP (15 μM); DE: infected cells treated with DE (15 μM). *** *p* < 0.001, ** *p* < 0.01 and * *p* < 0.05 compared to Infected Control; # *p* < 0.1 compared to Control. Data were analyzed by ANOVA, followed by Tukey’s post hoc test, using GraphPad Prism 5.0. (**A**) TNF-α production in pg/mL; (**B**) IL-10 production in pg/mL; (**C**) IL-1β production in pg/mL; (**D**) MFI percentage in NLRP3+ cells.

TNF-α, IL-10, and IL-1β cytokine production was evaluated during *M. smegmatis* infection of murine macrophages (Raw 264.7) to investigate the immunomodulatory activity of CP and DE during *M. smegmatis* infection. The results showed that there was an increase in TNF-α, IL-1β, and IL-10 production 24h after the infection by *M. smegmatis* (MOI 1:1) compared to the control group (Figure 9A–C). In this context, treating infected cells with CP (15 μM) led to a reduction in TNF-α (34.07%) and IL-1β (69.35%) cytokine production (Figure 9A,C). Similarly, treating infected cells with DE (15 μM) resulted in less TNF-α (56.94%) and IL-1β (59.82%) production (Figure 9A,C). Although the infection increased IL-10 production, the treatments did not alter its levels (Figure 9B). In addition, it was investigated whether *M. smegmatis* infection of murine macrophages would promote greater NLRP3 inflammasome expression and if the treatment with CP and its analog DE could modify this expression. The data showed that the infection of murine macrophages with *M. smegmatis* increased NLRP3 expression by 32.9% compared to the control group (Figure 9D). In this context, treatments with CP and DE (24 h) reduced NLRP3 expression by 29.12% and 39.81%, respectively (Figure 9D). Taken together, these data demonstrate the potential of CP and DE to act as anti-inflammatory molecules during mycobacterium infections.

### 3.5. M. tuberculosis Susceptibility Investigation

**Figure 10:**
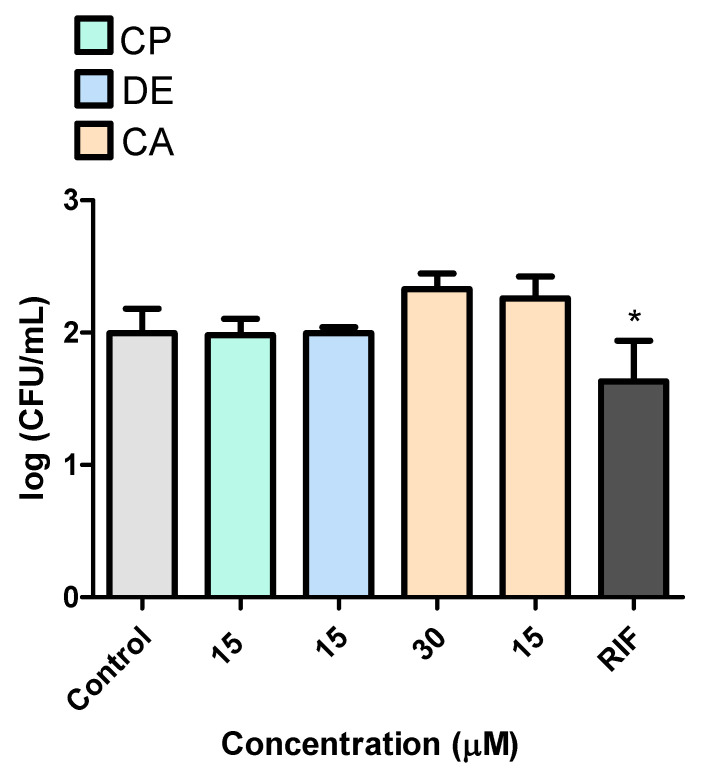
Evaluation of the effects of CP and its analogues on the viability of *M. tuberculosis* after 48 h incubation. Control: 2.5% DMSO-treated group. RIF: 0.03 μM. * *p* < 0.05 compared to the control group. Data were evaluated by ANOVA, followed by Dunnett’s post hoc test, using GraphPad Prism 5.0.

We initially determined the highest non-cytotoxic concentrations of CP, DE, and CA in RAW 264.7 macrophages in order to assess the susceptibility of *M. tuberculosis* to CP and its analogues. When tested against *M. tuberculosis*, CP, DE, and CA showed no inhibitory effect on bacterial growth over 48 h, indicating that the inhibitory concentration is greater than 15 μM for CP and DE and greater than 30 μM for CA (Figure 10). The reference antituberculosis drug RIF was evaluated at 0.03 μM as a positive control. Thus, RIF reduced the bacterial burden by 0.362 log_10_ CFU/mL after 48 h of treatment.

### 3.6. Infection Experiments Using Macrophages and M. tuberculosis

**Figure 11:**
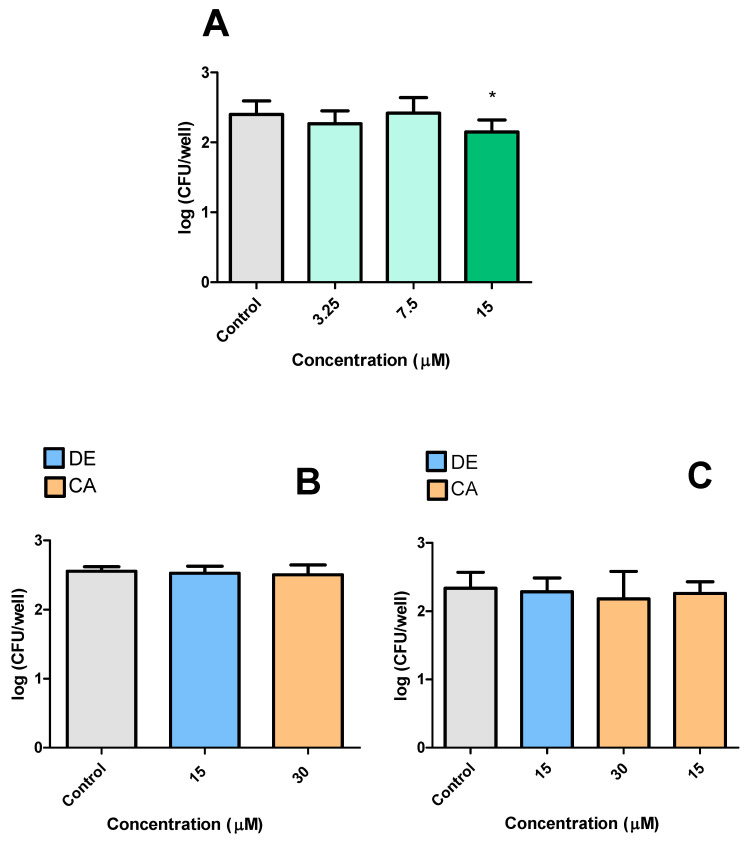
Effects of CP and its analogues in macrophages infected with *M. tuberculosis*. Incubation with CP for 24 h (**A**). Incubation with DE and CA for 24 h (**B**) and 48 h (**C**). Control: 0.5% DMSO-treated group. * *p* < 0.05 compared to the control group. Data were evaluated by ANOVA, followed by Dunnett’s post hoc test, using GraphPad Prism 5.0.

The first infection experiment investigated the effects of CP, DE, and CA on RAW 264.7 macrophages infected with *M. tuberculosis* (Figure 11). CP at 15 μM demonstrated an inhibitory effect after 24 h of incubation, resulting in a 0.250 log_10_ decrease in CFU counts (Figure 11A). Lower CP concentrations were subsequently tested, but showed no inhibitory effect. The effects of DE (15 μM) and CA (30 and 15 μM) were investigated at both 24 h (Figure 11B) and 48 h (Figure 11C). Based on previous viability studies of RAW 264.7 cells exposed to CP analogues, the treatment duration could be extended to 48 h for infected macrophages. However, neither DE nor CA showed inhibitory effects at either timepoint.

**Figure 12:**
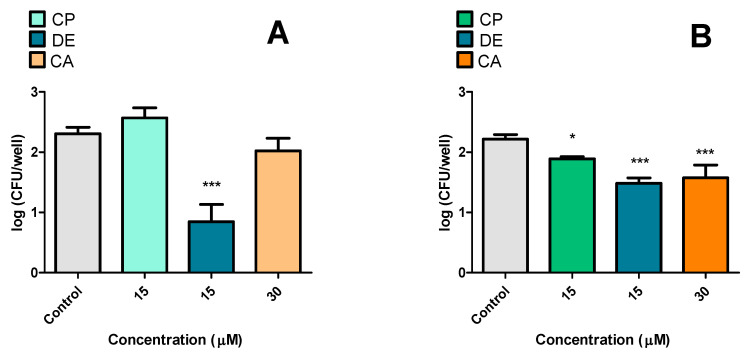
Effects of pre-incubation of CP and its analogues with RAW 264.7 infected with *M. tuberculosis.* (**A**): immediately after 3 h of infection, (**B**): 12 h after infection. Control: 0.5% DMSO-treated group. *** *p* < 0.001 and * *p* < 0.05 compared to the control group. Data were evaluated by ANOVA, followed by Dunnett’s post hoc test, using GraphPad Prism 5.0.

The second experiment aimed to analyze the effects of pre-incubating of RAW 264.7 cells with CP, DE, and CA prior to *M. tuberculosis* infection (Figure 12). The findings revealed that pre-incubation with DE at 15 μM significantly reduced bacterial loads (Figure 12A). It was observed that CP and DE at 15 μM, and CA at 30 μM, demonstrated significant reductions in CFU counts after 12 h of infection (Figure 12B).

### 3.7. Molecular Docking Simulations

The CP and DE compounds were subjected to molecular docking screening in the COX-1 (AlphaFold Model) and COX-2 (PDB: 6COX) enzymes. Redocking was performed prior to performing the molecular docking simulation in order to validate the targets under study. The submitted target comprised the COX-2 enzyme in complex with the selective SC-558 (COX-2) (PDB: 6COX) inhibitor, since it presents a co-crystallized ligand. Redocking is a simulation that evaluates whether there are structural differences between the co-crystallized ligand and its most stable pose, as well as assesses whether the molecular docking program is generating the poses correctly. Redocking is evaluated using the RMSD (root mean square deviation) value, with the appropriate value being 2.0 Å. For the target under study, it was observed that the RMSD value corresponded to 0.3121, validating the method under study.

The affinity score of the CP and DE compounds with the COX-1(AlphaFold Model) and COX-2 (PDB: 6COX) enzymes can be seen in Table 1.

**Figure 13:**
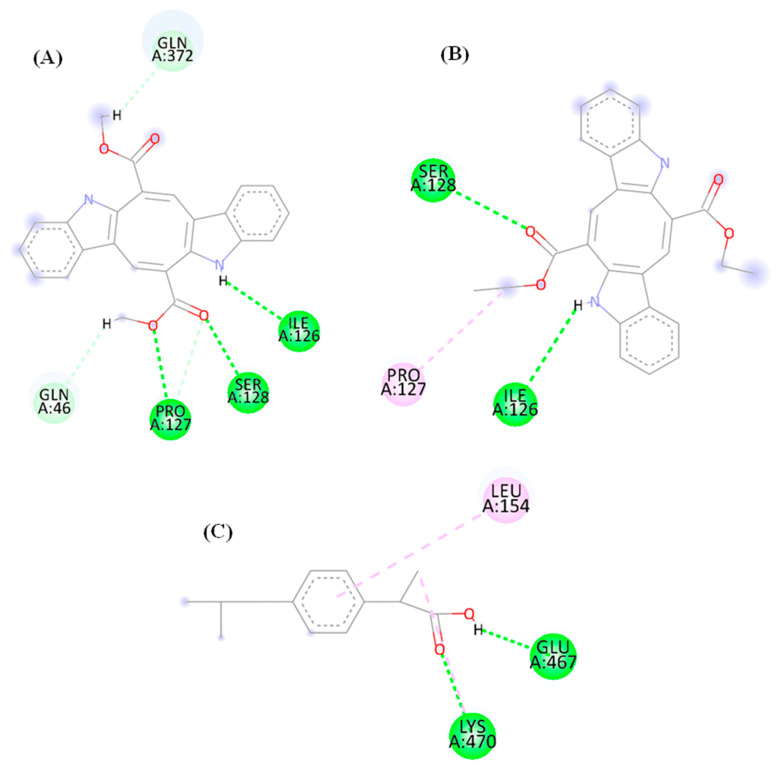
Molecular interaction maps of the CP (**A**) and DE (**B**) compounds and Ibuprofen (**C**). Interactions: Conventional hydrogen interaction (dark green dashed line), carbon–hydrogen interaction (light green dashed line), alkyl interaction (light pink dashed line). Residues: Gln (Glutamine), Ile (Isoleucine), Ser (serine), Pro (Proline), Glu (Glutamic Acid), and Lys (Lysine).

**Figure 14:**
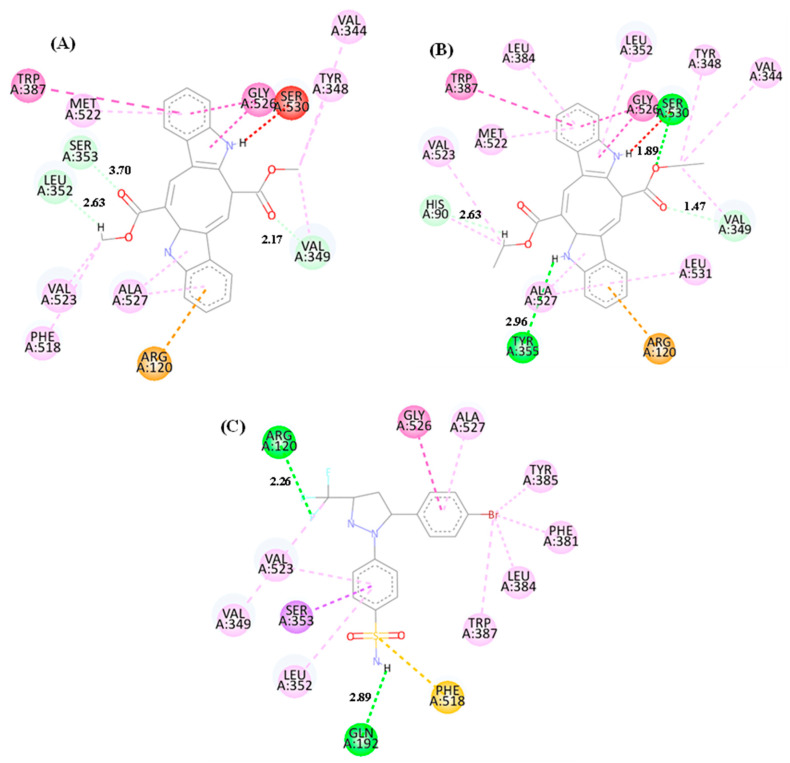
Molecular interaction maps of the CP (**A**) and DE (**B**) compounds and the PDB SC-558 ligand (**C**). Interactions: Conventional hydrogen interaction (dark green dashed line), carbon–hydrogen interaction (light green dashed line), alkyl and pi–alkyl interaction (light pink dashed line), Pi–Pi T-shaped and Pi–Stacked Amide interaction (dark pink dashed line), Pi–sigma interaction (purple), Pi–sulfur interaction (orange dashed line), unfavorable interaction (red dashed line). Residues: Val (Valine), Tyr (Tyrosine), Ser (serine), Val (Valine), Gly (Glycine), Trp (Tryptophan), Met (Methionine), Leu (Leucine), Ala (Alanine), Arg (Arginine), His (Histidine), Phe (Phenylalanine) and Gln (Glutamine).

According to Table 1, it is possible to observe that the CP and DE compounds presented negative binding energy values for the targets under study, indicating that interaction occurred. Greater affinity for the target COX-1 (AlphaFold Model) was observed, since the compounds under study presented lower binding energy values when compared to the control compound Ibuprofen, with the DE compound presenting the highest affinity corresponding to −138.966, while the CP compound presented the second highest affinity with a score value corresponding to −126.619, and the positive control Ibuprofen presented score values of −91.114. It was observed that the compounds presented similar binding energy values for the target COX-2 (PDB: 6COX) to those presented by the PDB ligand SC-558, with this being the most stable compound with binding energy values corresponding to −144.343, while the DE compound presented the second lowest energy value with a score corresponding to −137.589. CP presented a score value corresponding to −132.423. In addition to the energy representation, it is possible to visualize the molecular interaction maps of the CP and DE compounds with the COX-1 (AlphaFold Model) and COX-2 (PDB: 6COX) enzymes in Figure 13 and Figure 14.

### 3.8. Molecular Interaction Maps of the CP and DE Compounds with the COX-1 Enzyme AlphaFold Model

A greater number of hydrogen bond interactions were observed in the molecular interaction maps of the CP (Figure 13A) and DE (Figure 13B) compounds and positive control Ibuprofen (Figure 13C), with the target COX-1 obtained with an Alpha Fold model (Figure 13). Conventional hydrogen interactions (dashed line in dark green) were observed for the CP compound (Figure 13A) through Ile 126, Ser 128, and Pro 127 residues, which were observed in the amine and ester groups, as well as carbon–hydrogen interaction (dashed line in light green) through Gln 46 and Gln 372 residues, which were mainly observed in the acid group. A contribution of the ester groups and the amine group in forming conventional hydrogen interactions (dashed line in dark green) was observed for the DE compound (Figure 13B), which were established by Ser 128 and Ile 126 residues. A hydrophobic interaction of the alkyl type (dashed line in pink) was also observed through the Pro 127 residue. No similar interactions were observed between the residues observed and the compounds under study with the positive control Ibuprofen (Figure 13C), which may indicate that the compounds interact in a different site.

### 3.9. Molecular Interaction Maps of the CP and DE Compounds with the COX-2 Enzyme (PDB: 6COX)

A greater number of hydrophobic interactions were observed (Figure 14) in the molecular interaction maps of the CP (Figure 14A) and DE (Figure 14B) compounds and the Ibuprofen PDB ligand (Figure 14C) with the target COX-2 (PDB: 6COX). Carbon–hydrogen interactions (dashed line in light green) were observed for the CP compound (Figure 14A) through Ser 353, Leu 352, and Val 349 residues, which were observed in the acid and ester groups. The hydrophobic interactions were of the Pi–Pi T-shaped and Pi-stacked amide type (dark pink dashed line) through Gly 526 (2 interactions) and Trp 387 residues, as well as alkyl and Pi–alkyl interactions (light pink dashed line) through Met 522 and Ala 527 residues (2 interactions), which were observed in the aromatic rings of the structure. Other hydrophobic interactions of the alkyl and Pi–alkyl type were visualized in the methyl groups (CH3) of the ester group through Val 344, Val 349, and Tyr 348 residues, as well as in the Hydrogen atoms (H) of the acid group through Val523 and Phe518 residues. It was also possible to visualize the Pi–cation type iteration in the aromatic rings (orange dashed line) through the Arg120 residue. Furthermore, an unfavorable interaction (red dashed line) was observed through the Ser 530 residue on the hydrogen atoms (H) of the amine group. Conventional hydrogen interactions (green dashed line) were observed for the DE compound (Figure 14B) through the Tyr 355 and Ser530 residues, which were observed in the amine and ester groups, as well as carbon–hydrogen interactions (light green dashed line) through the His90 and Val349 residues, which are present on the carbon atoms (Figure 14C) of the ethyl group. Other interactions visualized included hydrophobic interactions observed in the aromatic ring groups and in the ethyl groups, these being of the Pi–Pi T-shaped and amide Pi-stacked interaction type (dark pink dashed line), established by Gly 526 and Trp 387 residues, and alkyl and Pi–alkyl iterations (dashed line in light pink) were observed through Val344, Tyr348, Leu352, Leu384, Met522, Val523, His90, and Ala527 residues (two interactions). Other interactions visualized were of the Pi–cation type (dashed line in orange) through the Arg120 residue and an unfavorable interaction (dashed line in red) through the Ser530 residue. Similar interactions were observed between the compounds under study and the PDB SC-558 ligand comprising Val523, Ala527, and Val 349 residues referring to alkyl and Pi–alkyl interactions (dashed line in light pink) and to Gly526 residue referring to amide–Pi stacked interactions (dashed line in dark pink).

### 3.10. Cytotoxicity Evaluation of CP, DE, and CA on Vero E6 and HepG2 Cell Viabilities and on A. salina Survival

**Figure 15:**
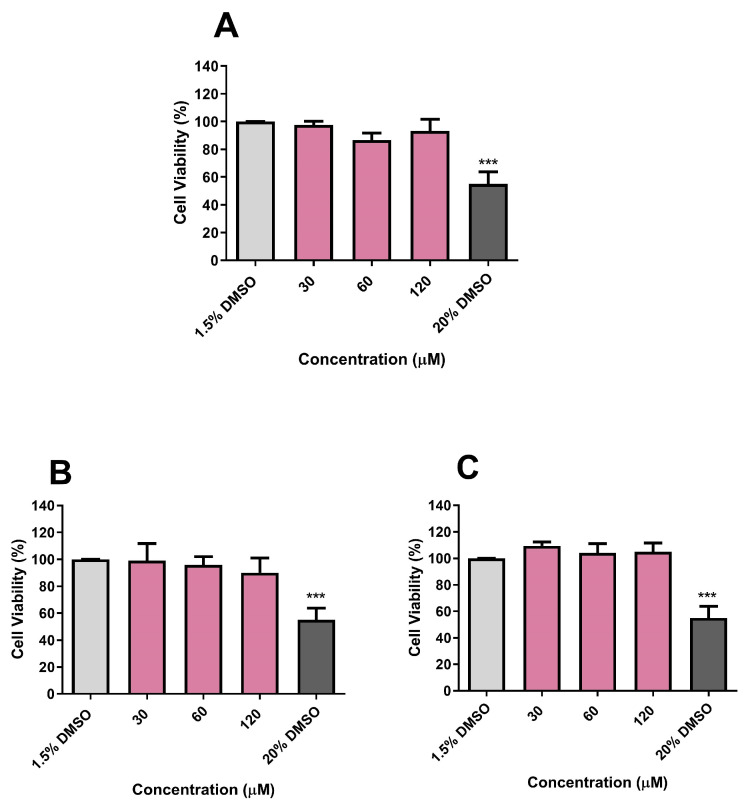
Effects of CP (**A**), DE (**B**), and CA (**C**) on Vero E6 viability after 24 h incubation. Control: 1.5% DMSO-treated group, considered as 100% of cell viability. *** *p* < 0.001 compared to the control group. Data were evaluated by ANOVA, followed by Dunnett’s post hoc test, using GraphPad Prism 5.0.

**Figure 16:**
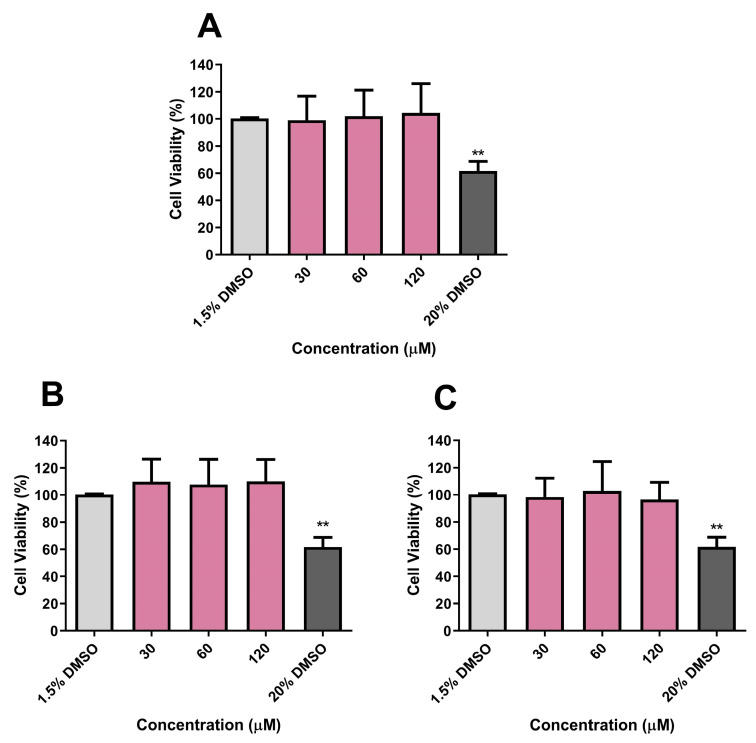
Effects of CP (**A**), DE (**B**), and CA (**C**) on HepG2 viability after 24 h incubation. Control: 1.5% DMSO-treated group, considered as 100% of cell viability. ** *p* < 0.01 compared to the control group. Data were evaluated by ANOVA, followed by Dunnett’s post hoc test, using GraphPad Prism 5.0.

After determining the immunomodulatory and antitubercular effects of CP, DE, and CA, the cytotoxic effects of these drug candidates were evaluated in the Vero E6 and HepG2 cell lines. Cytotoxicity experiments conducted at concentrations ranging from 30 to 120 µM demonstrated that CP, DE, and CA did not significantly reduce cell viability in either cell line over a 24 h period (Figure 15 and Figure 16). DMSO at a concentration of 20% was used as a positive control during cytotoxicity experiments.

**Figure 17:**
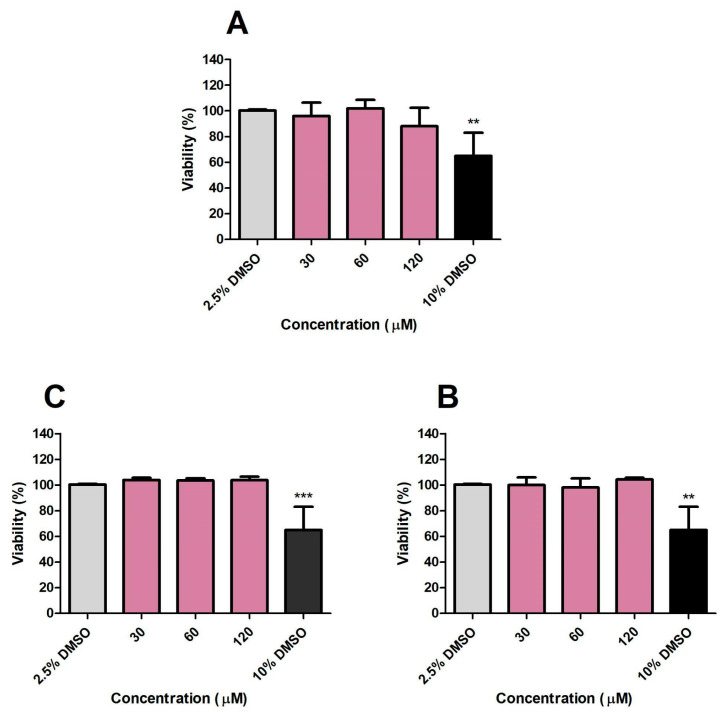
Effects of CP (**A**), DE (**B**), and CA (**C**) on *A. salina* survival after 24 h incubation. Control: 2.5% DMSO-treated group considered as 100% of *A. salina* survival. *** *p* < 0.001 ** *p* < 0.01 compared to the control group. Data were evaluated by ANOVA, followed by Dunnett’s post hoc test, using GraphPad Prism 5.0.

CP, DE, and CA were evaluated for potential toxic effects using brine shrimp (*A. salina*). Toxicity experiments conducted across concentration ranges from 30 to 120 µM demonstrated that CP, DE, and CA did not affect nauplii survival rates over a 24 h period (Figure 17). A 10% DMSO treatment was included as a positive control for mortality.

We then estimate that the IC_50_ values are higher than 120 µM for both eukaryotic cell lines and *A. salina*. The selectivity indexes are estimated to be higher than eight based on the concentration of 15 µM used in efficacy assays (macrophage infections). These results reinforce the potential of CP, DE, and CA for further preclinical development stages.

## 4. Discussion

The *M. tuberculosis* H37Ra and *M. smegmatis* mc^2^155 bacterial strains were used in this study. Considering the biosafety standards for research with biological agents, the H37Ra strain of *M. tuberculosis* may serve as an agent used in early drug discovery [31], and only slight differences in drug susceptibility have been observed between H37Ra and H37Rv strains [32]. The use of *M. smegmatis* in test development provides an effective preliminary model for evaluating drug activity against *Mycobacterium* spp. [33]. Genomic comparisons between *M. smegmatis* and *M. tuberculosis* reveal that these microorganisms share several characteristics, making *M. smegmatis* a valuable model for studying mycobacterial biology [34].

Macroalgae, or seaweed, contain bioactive compounds such as vitamins, minerals, pigments, and polysaccharides that can modulate the immune system [35]. Among the compounds derived from algae, ulvan, a sulfated gelling polysaccharide extracted from *Ulva ohnoi*, has demonstrated the ability to modulate the immune response in RAW 264.7 macrophages LPS-stimulated, promoting an increase in IL-1β, IL-6, and IL-10 cytokines [36]. Another alga with recognized immunomodulatory potential is *Gelidium amansii*, a red alga cultivated on the northeast coast of Taiwan, which promotes an increase in cell proliferation and the production of nitric oxide, TNF-α, IL-1β, and IL-6 in RAW 264.7 macrophages [37].

The present study evaluated the anti-inflammatory and immune-promoting effects of CP and its analogues in RAW 264.7 macrophages infected with *Mycobacterium* spp. Previous studies have demonstrated CP’s diverse biological activities, including spasmolytic effects in guinea pig ileum [38] and antinociceptive and anti-inflammatory activities in capsaicin-induced ear edema and carrageenan-induced peritonitis models. The antinociceptive effect was found to be dependent on α2-adrenoceptors and 5-HT3 receptors [39,40]. Additionally, *Caulerpa mexicana* extracts have shown the ability to reduce pro-inflammatory cytokines such as IL-6, IL-12, TNF-α, and IFN-γ in a zymosan-induced peritonitis mouse model [41].

Our results demonstrate that CP, DE, and CA inhibited the intracellular growth of *M. smegmatis* in RAW 264.7 macrophages. Furthermore, CP and DE modulated production of IL-1β and TNF-α during *M. smegmatis* infection. IL-1β is a key mediator of inflammation and plays an important role in host resistance by promoting immune activation to prevent TB development [42]. The modulating effects of CP or its extract on IL-1β in macrophages have been previously documented in several studies. Yoojam et al. [43] reported that *Caulerpa lentillifera* extract exhibited anti-inflammatory effects in LPS-stimulated RAW 264.7 cells by decreasing IL-6, TNF-α, and IL-1β production. Zeng et al. [44] demonstrated that *Caulerpa chemnitzia* polysaccharides promote upregulation of IL-1β, IL-6, and TNF-α in macrophages through the succinate pathway. Additionally, Cuomo et al. [17] showed that cells pretreated with CP following incubation with *H. pylori* culture filtrate or a non-formylated peptide derived from *H. pylori* exhibited reduced expression of IL-8, IL-6, and TNF-α cytokines compared with untreated control.

TNF-α plays a vital role in both protection against and promotion of *M. tuberculosis* infection, influencing immune cell activation, granuloma formation, and the inflammatory response. However, it may also cause chronic inflammation and tissue damage [45]. The inhibitory effect of CP on TNF-α was previously demonstrated in a murine model of peritonitis and ulcerative colitis, in which CP doses of 40 mg/kg reduced IFN-γ, IL-6, and TNF-α levels [16]. Lu et al. [46] demonstrated that *Caulerpa microphysa* ethyl acetate extract reduced IL-1β, IL-6, IL-8, and TNF-α production and promoted collagen homeostasis by inhibiting skin inflammation in an LPS-stimulated human epidermal keratinocyte cell line. Reports of CP modulating IL-1β and TNF-α in previous studies corroborate the findings of this work, confirming that CP could reduce proinflammatory cytokine levels, which might help control chronic inflammation in TB infections.

From the development of the infection model with *M. smegmatis*, we observed that CP and DE reduced NLRP3 inflammasome levels during infection. NLRP3 inflammasomes are multiprotein complexes which play a fundamental role in the body’s immune response to mycobacterial infection by providing a platform for caspase-1 activation. Activated caspase-1 is responsible for cleaving pro-inflammatory cytokine precursors into their active forms, such as IL-18 and IL-1β. NLRP3 inflammasomes function to activate IL-1β and IL-18 during *M. tuberculosis* infection, contributing to coordinate the immune response to control TB infection [47,48]. Our findings align with this established mechanism, as we observed that CP and DE reduced both IL-1β levels and NLRP3 inflammasome assembly in the *M. smegmatis* infection model.

COX-2 and the NLRP3 inflammasome are both involved in the pathogenesis of inflammatory diseases. After conducting docking simulations with the COX enzyme, our results suggested that CP and DE have the potential to interact with both COX-1 and COX-2 isoforms. COX is an enzyme responsible for the production of prostaglandins, chemicals that play a key role in inflammation, making it a crucial target for studying the anti-inflammatory and antipyretic activity of new compounds. This is because antipyretics reduce fever and pain by inhibiting COX. The ability of CP and DE to inhibit this enzyme enhances their anti-inflammatory effects, which could be a valuable characteristic for a new agent used for treating TB in the future [49].

Cells pretreated with CP, DE, and CA and subsequently infected with *M. smegmatis* and *M. tuberculosis* showed reduced bacterial internalization in macrophages. Macrophages play an important role in *M. tuberculosis* infection progression, as this bacterium multiplies intracellularly and can thereby escape host defense mechanisms [49]. While the mechanisms by which CP, DE, and CA influence bacterial internalization in this study remain unknown, a study by Abílio et al. [15] showed the ability of CP and its analogues in reducing Herpes simplex virus type 1-induced cytotoxicity in Vero E6 cells.

The results of this study suggest that CP, DE, and CA may act as immunomodulatory agents in *Mycobacterium* spp. infection in RAW 264.7 cells. The RAW 264.7 cell line is an effective in vitro model for murine macrophages due to its natural ability to perform both phagocytosis and pinocytosis, making it essential for studying host–pathogen interactions [50,51]. However, it is important to highlight that the development of in vivo assays may provide greater precision and enable analyzing interactions with other biological pathways. In this context, creating infection models in mice is essential to better understand the mechanisms by which CP, DE, and CA influence the internalization of *M. smegmatis* and *M. tuberculosis*.

The development of toxicity tests is vital for developing new drugs. Drug toxicity can manifest in various ways and contributes to the high cost of drug development, particularly when not identified until late-stage clinical trials or post-marketing. In vitro models are useful tools for evaluating preliminary toxic effects in specific cell types, as they offer a rapid and cost-effective screening method [52].

The monkey kidney cell line Vero E6 has been widely used in toxicological research. In the present work, we observed that CP, DE, and CA did not affect the viability of Vero E6. Abílio et al. [15] observed that CP and its analogues did not affect Vero E6 viability when exposed to concentrations ranging from 200 to 1800 µM for 72 h. Macedo et al. [53] evaluated the antiviral capacity of CP against herpes simplex virus type 1 and conducted cytotoxicity tests with Vero E6. After exposing cells to CP at concentrations ranging from 50 to 1000 µM for 72 h, they observed no effect on Vero E6 viability. Esteves et al. [54] evaluated the antiviral potential of CP against the Chikungunya virus and found that Vero E6 cells exposed to 589.3 µg/mL of CP maintained their viability. The results obtained in this study, together with those previously described [15,53,54], suggest that CP, DE, and CA do not exhibit renal toxicity and are potential candidates for further toxicological studies.

The liver is a primary target for systemic toxicity caused by chemicals, making the development of tests to investigate the hepatotoxic potential of drug candidates essential [55]. In this work, we used the HepG2 cell line to evaluate the hepatic toxicity of CP, DE, and CA. HepG2 cells are derived from human hepatocellular carcinoma, and their use in in vitro testing to investigate the hepatotoxicity of anti-TB drug candidates has been validated by previous studies [56,57]. Our results showed that CP, DE, and CA did not affect HepG2 cell viability at the tested concentrations over 24 h. While no previous studies have investigated CP’s hepatotoxic potential in HepG2, Abdelrheem et al. [58], demonstrated that CP exhibited anticancer activity in HepG2 cells at concentrations ranging from 15.80 to 500 µg/mL over 24 h. Our results importantly suggest that CP, DE, and CA may be suitable candidates for further efficacy and safety testing in murine models.

The *A. salina* toxicity assay is widely used in preliminary toxicity studies and is particularly valuable in early-stage drug development for evaluating compound safety [59]. In our study, CP, DE, and CA did not affect brine shrimp survival over 24 h at the tested concentrations. While previous studies have examined marine compounds and extracts using *A. salina* toxicity assays, such as Ara et al. [60], who found that *C. racemosa* aqueous extract showed high lethal activity (IC50 value < 70 µg/mL), and Nofiani et al. [61], who reported that dried *C. lentifera* extract was lethal to *A. salina* nauplii and fresh extract had no effect on nauplii survival, our study is the first to demonstrate that purified CP and two analogues show no toxicity in this model system.

## 5. Conclusions

**Figure 18:**
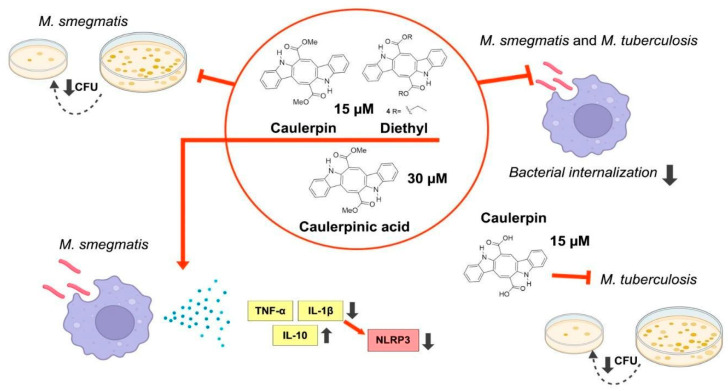
Summarization of the effects of CP, DE, and CA in RAW 264.7 macrophages infected with *Mycobacterium* spp.

In conclusion, this study demonstrated that CP and two additional analogues (DE and CA) exhibited significant anti-inflammatory and anti-tubercular activities in RAW 264.7 macrophages infected with both *M. smegmatis* and *M. tuberculosis* H37Ra (Figure 18). Their effects were evidenced by modulating pro-inflammatory cytokine production and reducing the bacterial burden. Our findings also demonstrate that CP, DE, and CA possess immunomodulatory properties which may influence the internalization of mycobacterial bacilli in macrophages (Figure 18). Further pharmacological and immunological studies are needed to elucidate the precise mechanisms underlying these immunomodulatory effects. This evaluation of CP against *M. tuberculosis* in RAW 264.7 macrophages indicates its potential as a promising compound for further host-mediated developmental studies. Overall, these results make a significant contribution to the pharmacological understanding of marine natural products and its derivative molecules as host-directed drug candidates against infectious diseases. However, developing additional tests to validate the results presented in this study is necessary. In this regard, conducting tests to explore the combinatorial effects of CP with drugs used in treating TB would strengthen the discussion of CP as a promising investigational compound for HDT therapy. Finally, developing tests using virulent *M. tuberculosis* strains and murine TB models may provide better validation of our results, as well as enable evaluation of the pharmacokinetic profile and any potential toxic effects.

## Figures and Tables

**Table 1:** Binding energy values of the compounds under study according to the MolDock Score algorithm for the COX-1(AlphaFold Model) and COX-2 (PDB: 6COX) enzymes.

Compounds	COX-1 (AlphaFold Model)	COX-2 (PDB: 6COX)
CP	−126.619	−132.423
DE	**−138.966**	−137.589
Positive Control/PDB Ligand	−91.114	**−144.343**

Legend: The compound with the lowest energy is highlighted in bold. Positive Control: Ibuprofen; PDB-ligand: SC-558.

## Data Availability

The original contributions presented in this study are included in the article. Further inquiries can be directed to the corresponding author.
